# The Athletic Intelligence Quotient and performance in the National Basketball Association

**DOI:** 10.3389/fpsyg.2023.1197190

**Published:** 2023-07-14

**Authors:** Scott R. Hogan, Daniel Taylor, R. Thomas Boone, James K. Bowman

**Affiliations:** ^1^Strength Coach Scott LLC, Pittsburgh, PA, United States; ^2^Charlotte Hornets, Charlotte, NC, United States; ^3^Department of Psychology, University of Massachusetts Dartmouth, North Dartmouth, MA, United States; ^4^Great Neck Public Schools, Great Neck, NY, United States

**Keywords:** NBA, NBA draft, basketball, intellectual ability assessment, AIQ, Athletic Intelligence, athletes, cognitive abilities

## Abstract

Prior to selecting an NBA player, teams consider multiple factors, including game film and tests of agility, strength, speed, anthropometry, and personality. In recent years, as the other major professional sports have begun to place greater emphasis on the measurement of cognitive abilities, so too have representatives in the NBA. In this study, the predictive validity of an empirically-supported measure of cognitive ability (AIQ) was examined vis-à-vis performance outcomes in the NBA. Specifically, AIQ scores were obtained from 356 NBA prospects prior to their draft between 2014 and 2019. The players’ professional status and subsequent performance were assessed through composite and isolated NBA statistics. ANOVAs demonstrated that there were significant differences between NBA and non-NBA players, and subsequent independent samples *t*-tests revealed that NBA players had significantly higher AIQ scores than non-NBA players for 3 out of 4 factors and the Full Scale AIQ Score. Additionally, using hierarchical multiple regression analyses, it was demonstrated that the AIQ predicted some modest statistically significant relationships with multiple NBA stats (e.g., Player Efficiency Rating, Effective Field Goal Percentage), after controlling for the impact of draft placement. While the effect sizes for these differences and relationships were somewhat small, such findings are consistent with sport analytics and the restricted range when evaluating professional athletes. Given the expanding role of analytics and cognitive assessment in the NBA, the potential importance of the AIQ is considered in the draft process.

## Introduction

Basketball ranks among the top sports in both the United States and the world in terms of participation rate (Hulteen et al., 2017). In addition to being one of the most popular sports, basketball is also one of the most profitable. Each of the 30 organizations in the NBA is valued at greater than $1 billion. As of 2020, player contracts (12–15 per team) totaled approximately $100 million annually, with a plurality of the money often being devoted to superstars, who may account for up to 35% of a team’s salary cap each. Success is typically measured in wins and losses, and never was that clearer than in 2019 when the value of the Toronto Raptors increased by an incredible 25% following their first NBA championship (Badenhausen, 2020).

Identifying talent early in players’ careers is critical for NBA organizations, and the draft offers each team an annual opportunity to improve their roster and build for the future. Navigating the inexact science of player selection is of premium interest to scouts, general managers and other front office personnel. To this end, organizations spend a great deal of time and resources finding novel ways to predict the future abilities of both young athletes and free agents in an effort to successfully build and bolster their rosters.

### College basketball performance

Players’ college basketball performance is perhaps the most readily available source of data for NBA teams to consider. In addition to reviewing game statistics, NBA scouts and other team representatives attend games and watch detailed footage of players to try to project how they might fare in the NBA. Research on the effects of college basketball performance on subsequent NBA performance suggests that there is good reason to place emphasis on their past performance. For instance, according to Moxley and Towne (2015), the “percent of college win shares” in a player’s final season was significantly correlated with NBA win shares in each of a player’s first three seasons in the NBA, with moderate correlation coefficients.

Although this factor appears to be a significant predictor of subsequent NBA performance, one of the challenges of quantifying college performance is determining the impact of college quality (Moxley and Towne, 2015). That is, some college teams and conferences play at a higher level than others. Thus, a player averaging 25 points per game for Duke University is likely to be different from a player who averages 25 points per game for Iona College.

Another challenge when evaluating prior college basketball performance is that there may be a limited sample of game play to evaluate. Although some players play multiple seasons in college, basketball is unique in that players can declare for the NBA draft at 19 years old. Therefore, some of the best players in a given draft year may only have a year or two of college basketball experience for teams to consider. In fact, out of the 58 draft picks in the 2022 NBA draft, 21 played only 1 year of college basketball (Breaking Down the NBA Draft by College Experience, 2022).

### Assessment of physical skills and anthropometry

The NBA Draft Combine is an annual event designed to examine the league’s top prospects by testing athletes’ anthropometry, strength, agility, and shooting skills, with teams also conducting medical examinations and interviews with prospective players. The current format for the Combine was developed by the National Basketball Coaches Association (NBCA) testing committee in 2000, allowing for standardized performance testing and leading to more efficient talent evaluation (Milan et al., 2019). Each drill or measurement was selected to represent the most valid and reliable assessment available of basketball performance and the physical qualities that underpin success in this sport (Teramoto et al., 2018).

In the past 20 years, the NBA Combine has become an important date for agents, players, and front office staff alike. Anecdotal reviews of players are highly prevalent, particularly in the media, and physical metrics and performance can generate considerable interest. However, empirical research on the predictive validity of these measurements is inconsistent. For instance, Ranisavljev et al. (2020) found that NBA Combine physical assessments showed only low to moderate correlations with basketball performance variables within a player’s first season. The highest correlation was reported between upper body strength and the number of rebounds and blocked shots a player would garner. Similarly in 2015, Moxley and Towne reported that physical anthropometry played an insignificant role in success in the NBA. Further, in a detailed analysis of the NBA Combine metrics from 2000 to 2005, Teramoto et al. (2018) indicated that only the anthropometric data, labeled as ‘length-size’ was a significant predictor of on-court performance in first and third-year players.

Conversely, others have found a variety of significant relationships between the results of physical testing and future performance. Specifically, low body fat percentage and leaping ability showed potential to predict defensive abilities in players. Additionally, lateral quickness was an important indicator of the ability to steal the ball from opposing players (Huyvaert et al., 2015).

One significant factor that limits the discriminability of physical assessments, however, is range restriction, as there tends to be great parity in physical skills when comparing the top players in sports (Bergkamp et al., 2019). This undoubtedly makes comparisons of physical skills/attributes among potential lottery picks more challenging. However, this may be less problematic when looking at a broader and more diverse range of players. In a recent study by Cui et al. (2019), comparisons were made among drafted and undrafted players who participated in the NBA Draft Combine tests from 2000 to 2018. The drafted players from all five positions had significantly higher scores than undrafted players in height, wingspan, vertical jump height and reach, line agility, and three-quarter sprint test (Cui et al., 2019).

Although physical assessments and anthropometry certainly represent one piece of the puzzle, in terms of success in the NBA, these metrics alone clearly do not account for differences in performance. For instance, using only these kinds of metrics would not explain outliers such as Kevin Durant, who was unable to perform even a single repetition on the upper body strength test (bench press) yet went on to average 4.5 rebounds and nearly one block a game during his rookie year (Gleeson, 2017). More impactful still, Durant is considered a generational talent in his own right and is in the NBA’s top 10 all-time player efficiency ratings (PER), per Basketball Reference (2020).

As the case of Durant illustrates, a player’s success in the league is driven by more than pure athleticism and feats of strength. While the current research does show some statistically significant relationships between certain physical tests and measurements and subsequent NBA performance, there remains a lack of consensus on which physical capacities tested can spotlight career success. Moreover, there appears to be a significant amount of variance in NBA performance that may be explained by other factors.

### Psychological assessment in sports

The psychological attributes of athletes represent an area that has consistently been acknowledged but typically not measured with fidelity within the selection process. Research has been conducted on various aspects of an athlete’s psychological makeup for over 40 years. As early as the late 1970’s, investigators such as Morgan and his associates pioneered work in the profiling of an athlete’s mood state (Nagle et al., 1975; Morgan and Johnson, 1978; Morgan, 1985). Measures such as mood profiling are typically administered as a way to quantify the training response of an athlete. While mood has been shown to be a performance predictor, it must be noted that the time frame of assessment holds a major influence, which limits the utility of the results (Terry et al., 2005).

Other common psychological tools used in training contexts among athletes are the Emotional Recovery Questionnaire, the Total Quality Recovery Scale, the Daily Analyses of Life Demands for Athletes, the Recovery-Stress Questionnaire, the Acute Recovery and Stress Scale, the Short Recovery and Stress Scale, and the Multi-Component Training Distress Scale (Nässi et al., 2017). Each of these tests has shown the capacity to deliver valuable information for athletes and coaches, but they are all simply monitoring assessments used for observing phenomena such as changes in mood, emotions, levels of perceived stress, recovery, and sleep quality. While these tools may be helpful for guiding training interventions, psychometrics from tools such as these do not aid in predicting long term achievements or differentiating between elite and average athletes.

Despite their frequent use in the athletic realm, many psychological assessments suffer from issues of validity in a selection context, stemming from the fact that the tools available to most practitioners are based on self-reported measures. The limitations of self-reported assessments may be attributed to the unknown motivations behind how individuals choose to answer questions, including biases associated with social desirability and the consistency motif (Park et al., 2016). Though there are certainly informative and valid self-report measures of mood state, perceived stress, personality, and other psychological factors that have demonstrated utility in a variety of contexts, the use of such measures for selection purposes in sports, in particular, is more fraught.

### Measurement of cognitive functioning in sports

The relationship between cognitive skill and athletic performance has been an area of study for over 40 years. Investigations in this area have revealed that expertise in sport is underpinned by perceptual and cognitive skill as well as the capacity to execute effective patterns of movement. It has been reported that experts differ from novices across a spectrum of perceptual and cognitive measures such as enhanced capacity in recalling and recognizing patterns of play as well as a heightened ability to use advanced visual cues to anticipate an opponent’s actions (Williams et al., 2003). Additionally, elite athletes have shown a significant advantage in aspects of executive functioning (Jacobson and Matthaeus, 2014).

Within the field of cognitive assessment research in sports, the expert performance approach is performed in an environmentally valid context wherein displays of sport-specific skills are designed to simulate the context of sport. While athletes have shown increased cognitive output compared to non-athletes in this context, the athlete’s superior knowledge operating in this environment may confound the results (Voss et al., 2010). For instance, some measures may utilize real-life basketball scenarios in their assessment of cognitive functioning, thereby creating an uneven playing field based on factors such as experience and general basketball knowledge. This is an especially important limitation to consider in the selection process for the NBA, where players can potentially start in the league at the age of 19, with much skill development still to come. Ultimately, being able to distinguish between acquired sport knowledge and skills from more fundamental cognitive abilities is critical because it is the latter which allows athletes to develop and perform sport-specific skills (Voss et al., 2010).

By contrast to the expert performance approach, the cognitive component skill approach instead seeks to determine the relationships among specific cognitive variables, measured in a neutral context, and sport performance. In the expert performance approach, there has been considerable research indicating that multiple aspects of cognitive functioning affect sport performance. In fact, based on growing research in this area, some have argued that the cognitive domain may be a determining factor distinguishing elite athletes (i.e., “playmakers”) from non-elite athletes (Zaichkowsky and Peterson, 2018).

Perhaps the most well-known test of intelligence in sports is the Wonderlic Personnel Test, which provides a measure of general mental ability (Solomon and Kuhn, 2014). The Wonderlic focuses primarily on the measurement of vocabulary, reading comprehension, and mathematical ability, which are all learned skills. Although the Wonderlic’s areas of focus are pertinent in many fields, they have consistently demonstrated a lack of predictive validity vis-à-vis sport performance (Outtz, 2002; Mirabile, 2005; Berri and Schmidt, 2010). An unfortunate conclusion from these findings has been that intelligence may not be important in sports (Lehrer, 2009). Although not all intellectual abilities may be relevant to sport, some aspects appear to be critical to an athlete’s success.

### Cattell-Horn-Carroll theory

Historically, intelligence was considered to represent a single general factor referred to as “g;” however, research-backed contemporary theories now include multiple types of intelligence (Schneider and McGrew, 2018). Of all the competing theories, the Cattell-Horn-Carroll (CHC) theory of cognitive abilities has the most support for its foundational principles (Flanagan et al., 2013). Consisting of an evidence base that includes developmental, neurocognitive and factor analytic research, CHC has been investigated widely and utilized across a variety of fields (Schneider and McGrew, 2018).

Through its vast underpinning of empirical support, CHC theory has provided a foundation for widespread revisions of notable intelligence and academic achievement tests such as the 5th Edition of the Wechsler Intelligence Scales for Children (Alfonso et al., 2005; Flanagan et al., 2013). Up to 18 broad cognitive abilities have been identified within CHC Theory, each of which is composed of several narrow abilities (Flanagan et al., 2006). Grounded within the CHC theory, there appear to be several broad intellectual abilities that are germane to the world of athletics. Specifically, the four broad CHC abilities of Visual Spatial Processing, Long-Term Storage and Retrieval, Reaction Time, and Decision-Making are all bespoke for the demographic of sports (Bowman et al., 2020). Additionally, the application of CHC theory potentially provides a common language for all coaches, athletes, and practitioners to facilitate the discussion of an individual’s cognitive strengths and weaknesses. Through this polyglottic framework and empirical foundation, conclusions about an athlete’s intellectual makeup can be drawn with confidence. However, as yet CHC theory has not been applied to the assessment of elite basketball athletes.

### The Athletic Intelligence Quotient (AIQ)

The AIQ was developed to apply CHC theory to the assessment of elite athletes by measuring a range of specific cognitive abilities that facilitate an athlete’s capacity to optimally visualize their surroundings in real-time, learn and recall game information fluently, react quickly and accurately to stimuli, and sustain rapid decision making for extended periods (Bowman et al., 2020). According to the authors of the AIQ, Athletic Intelligence focuses on a specific subgroup of CHC abilities, namely visual spatial processing (Gv), learning efficiency (Gl), reaction time (Gt), and decision-making (Gs). A fundamental difference between the AIQ and other measures that assess general mental ability is that it does not include more academic, cognitive abilities such as verbal knowledge and quantitative reasoning, though the learning efficiency subcategory correlates broadly with the more standard measures of intelligence (Bowman et al., 2020).

Due to its ease of implementation, rigorous validation, and broad utility based on the foundations of CHC Theory, the AIQ has become a psychological assessment of choice for professional athletes. Since 2012, teams in the National Football League (NFL) and Major League Baseball (MLB), in particular, have utilized the assessment and research has been undertaken on its predictive validity in these sports. During 2015 and 2016, 146 NFL Scouting Combine prospects were administered the AIQ (Bowman et al., 2020). Scores from these players’ AIQ performance were then used to predict subsequent on-field performance. The results of this investigation showed that factors within the AIQ accounted for a statistically significant increase in the explanation of variance in position-specific game statistics, such as rushing yards per carry. Additionally, significance was also found for the overall rating of player success (or weighted career approximate value) over other factors like draft order. In a separate study, the utility of the AIQ was also demonstrated in a cohort of minor league baseball players, as scores on a variety of subtests such as reaction time and decision making showed a significant effect on both hitting and pitching success (Bowman et al., 2021).

Although the AIQ has been used in the NBA for nearly 10 years, this is the first formal research investigation into the relationships among AIQ factors and performance outcomes in the NBA. Through the application of the AIQ to a large population of both NBA and other professional basketball players, our goal was to follow the cognitive component approach to clarify the role that specific cognitive abilities play in elite basketball.

### Hypotheses

Considering the body of existing research on cognitive functioning and sport performance, we proposed the following hypotheses:

*H1*: NBA players would have significantly higher scores than non-NBA players (i.e., International, G League) on the 4 factors of the AIQ (i.e., visual spatial processing, long-term storage and retrieval, reaction time and decision-making) and the Full Scale AIQ Score (FS-AIQ).

*H2*: Undrafted players who made it to the NBA would have significantly higher scores than non-NBA players on the 4 factors of the AIQ plus the FS-AIQ Score.

*H3*: The 4 factors of the AIQ would account for a statistically significant increase in the explanation of variance in specific NBA basketball statistics (i.e., points per game, free throw percentage, turnovers per game) beyond draft round.

*H4*: The 4 factors of the AIQ would account for a statistically significant increase in the explanation of variance in composite NBA basketball statistics (i.e., Player Efficiency Rating, Effective Field Goal Percentage (eFG%), Passing Efficiency) beyond draft round.

## Methods

### Participants

Three hundred and fifty-six NBA prospects were administered the AIQ between 2014 and 2019 at the NBA Combine. Of these, 227 players have some NBA experience (labeled NBA-only) while 129 possess some professional basketball experience below the NBA level (non-NBA). The following position players were included in this study: PG (*n* = 97), SG (*n* = 85), SF (*n* = 72), PF (*n* = 67), C (*n* = 35). Participation in the study was voluntary. Data were collected from individual players with their written consent, as part of the standard NBA draft evaluation process. Additionally, only anonymized data were accessed and used for this study.

### Instruments

#### Athletic Intelligence Quotient

The AIQ is a cognitive ability assessment composed of 10 subtests. During the time frame of this study, it was computer-administered by a software program on a 10.1” Samsung Galaxy Tab, running the Android Operating System. The AIQ subtests are presented in a fixed, successive order, with audio/visual instructions, practice problems, and feedback provided before the start of each task. The administration time for the AIQ generally ranges from 35 to 38 min.

The AIQ was designed to register a Full Scale AIQ score as well as scores across four main CHC factors: visual spatial processing, reaction time, decision-making, and learning efficiency. Although 10 subtest scores can be interpreted, only the Full Scale AIQ Score and the 4 factors were analyzed and included in this study, in order to minimize Type I error.

Each of the AIQ tasks was designed to minimize the impact of language, culture, formal education, and proficiency with technology. Thus, responses are generally made by simply tapping the chosen response choice on the screen. For instance, on a measure of visual spatial processing, individuals are asked to manipulate/rotate images in their minds to see how they would look under different circumstances. In particular, examinees are presented with a given target shape and they must decide whether the shapes below it are the same (only rotated) or are different and would need to be flipped over to look the same. The players select the shapes by touching the ones that are the same. For detailed information about each of the subtests and cognitive abilities measured by the AIQ, please refer to Bowman et al. (2021).

### Procedures

The assessment protocol was briefly described before participants were asked to provide informed consent. This included their right to discontinue the assessment at any time. When the athletes arrived at the evaluation room, they were individually led to a station by a trained administrator who briefly explained the testing procedures. Next, an examiner initiated the computer program on the tablet for the participants and presented them with headphones for audio instructions.

### Statistical analysis

Statistical analysis was completed using de-identified players codes rather than names (e.g., NBA1 or NNBA23) to ensure that the results of the study were free from bias or subjective associations. All statistical analyses described were completed using SPSS (IBM).

## Results

A total of 356 professional basketball players were administered the AIQ, including 227 current or former NBA players and 129 players with no NBA experience, composed of a mix of international and G-League players. Additionally, of the 227 NBA players, 155 were drafted players while the remaining 72 players were undrafted but eventually played in the NBA.

### AIQ score differences for NBA drafted, NBA undrafted, and non-NBA players

Each AIQ factor is reported as a standard score configured like IQ, with a mean value of 100 and standard deviation of 15. Athletes’ AIQ scores were examined across the four broad CHC factors of visual spatial processing, reaction time, decision-making, and learning efficiency, utilizing a series of parallel univariate one-way ANOVAs as a function of non-NBA players, undrafted NBA players, and drafted NBA players. Given the AIQ scoring, values obtained from the players for all four factors were normally distributed, with the bulk of scores falling between 70 and 130. Table 1 shows the mean differences in scores between the three groups across the four broad CHC factors. The univariate ANOVA for visual spatial processing revealed no difference across the three groups, *F*(2,353) = 1.92, *p* = 0.149, *η^2^* = 0.011, but the remaining ANOVAs did yield group differences for reaction time, *F*(2,345) = 5.62, *p* = 0.004, *η^2^* = 0.032; decision-making, *F*(2,351) = 3.70, *p* = 0.026, *η^2^* = 0.021; and learning efficiency, *F*(2,353) = 4.75, *p* = 0.009, *η^2^* = 0.026; respectively.^1^ Specifically, in reaction time, drafted NBA players did better than both undrafted NBA and non-NBA players, who were not different from each other.

**Table 1:** Means and standard deviations for 4 CHC factors and full scale AIQ across non-NBA, undrafted NBA, and drafted NBA players.

	Visual spatial processing *M* (*SD*)	Reaction time *M* (*SD*)	Decision making *M* (*SD*)	Learning efficiency *M* (*SD*)	Full scale AIQ *M* (*SD*)
Non-NBA players	95.9 (10.02)	93.4^a^ (11.70)	97.9^a^ (12.32)	92.7^a^ (13.23)	95.2^a^ (8.40)
Undrafted NBA players	97.9 (8.92)	93.6^a^ (10.48)	100.2^a,b^ (10.68)	96.7^b^ (13.85)	97.32^a,b^ (7.42)
Drafted NBA players	98.1 (9.84)	97.6^b^ (11.04)	101.4^b^ (10.05)	97.4^b^ (12.92)	98.43^b^ (7.60)

^*^ Within each column, different superscripts denote groups that are significantly different from one another at *p* < 0.05.

Further, drafted NBA players had higher decision-making scores than non-NBA players, while undrafted NBA players were not different from either drafted NBA or non-NBA players. Finally, in learning efficiency, both undrafted and drafted NBA players, who were not different from each other, had significantly higher scores than non-NBA players. Not surprisingly, this pattern across the subscales was reflected in the Full Scale AIQ, which also demonstrated that the non-NBA players scored significantly lower than the drafted NBA players, with the undrafted NBA players between the two groups and not significantly different from either, *F*(2,353) = 6.16, *p* = 0.002, *η^2^* = 0.034.

### Relationship between NBA performance and AIQ

NBA performance statistics only exist for NBA players; thus, the non-NBA players were subsequently removed from the remaining analyses. Additionally, there were a number of players that only had a small amount of play, so rather than having their performance metrics unduly influence the results, all NBA players who played less than 10 games were removed from analysis, leaving the final number of NBA players at 182. Players were separated into three rounds based on draft pick: Round 1 (Pick 1–30), Round 2 (Pick 31–60), and Round 3 (undrafted). For each of the subsequent basketball performance statistics, a parallel hierarchical multiple regression was conducted with the performance statistic as the DV and the four AIQ subscales as predictor variables, after controlling for draft round.

The following basketball performance measures were analyzed in this series of hierarchical multiple regressions: average points scored per game (PTS), turnovers per game (TO), Free Throw Percentage (FT%), Player Efficiency Rating (PER), Effective Field Goal Percentage (eFG%), and Pass Efficiency. The first three measures are considered as performance metrics and the latter three as performance composites since they required additional calculations involving a number of factors. Table 2 presents the descriptive statistics and zero order correlations for these 6 performance measures with the four AIQ subscales.

**Table 2:** Descriptive statistics and zero-order correlations for performance metrics and AIQ factors (*N* = 182)^1^.

	*M*	*SD*	Visual spatial processing	Reaction time	Decision making	Learning efficiency
PTS	6.30	3.92	−0.09	−0.06	0.05	−0.02
Turnovers	0.84	0.55	−0.15^*^	−0.10^†^	−0.05	−0.04
FT%	71.2%	13.7%	−0.08	0.07	0.16^*^	0.17^*^
PER	11.34	3.78	0.07	0.02	0.19^**^	0.02
Pass efficiency	1.70	0.94	0.10	0.07	0.14^†^	0.07
eFG%	49.3%	7.8%	0.17^*^	13^†^	0.30^***^	0.12
Visual spatial Processing	98.52	9.97	–	0.37^***^	0.43^***^	0.33^***^
Reaction time	96.26	10.91		–	0.34^***^	0.35^***^
Processing speed	101.21	9.69			–	0.21^**^
Learning efficiency	96.98	13.02				–

^†^*p* < 0.10, ^*^p < 0.05, ^**^p < 0.01, ^***^p < 0.001.

^1^All the analyses were pulled from 182 players, but there were one or two cases of missing data at the analysis level. *N* ranged from 180 to 182 across all the analyses.

### Points and AIQ

The overall model for PTS as a function of draft pick round and the 4 subscales of the AIQ was significant, *F*(2,177) = 23.00, *p* < 0.001, *R^2^* = 0.21, but only draft round was a unique contributor, explaining 21% of the variance. This non-significant result for the 4 AIQ subscales, paired with the lack of any significant zero-order correlations between the 4 AIQ subscales, suggests that non-cognitive factors may account for much of the variance in points. The full statistics for this analysis are presented in Table 3.

**Table 3:** Hierarchical regression of performance metrics as a function of draft pick and AIQ factors.

Step and predictor variables	*R^2^*	∆*R^2^*	*sr^2^*	*β*	SEB
PTS					
Step 1	0.20^***^	0.20^***^			
Draft pick round					
Turnovers					
Step 1	0.16^***^	0.16^***^			
Draft pick round					
Step 2	0.18^***^	0.02^*^			
Visual spatial processing			−0.14	−0.14	0.004
FT%					
Step 1	0.02	0.02			
Draft pick round					
Step 2	0.05^*^	0.03^*^			
Learning efficiency			0.17	0.17	0.001

^†^*p* < 0.10, ^*^p < 0.05, ^**^p < 0.01, ^***^p < 0.001.

### Turnovers and AIQ

The overall model for turnovers as a function of draft pick round and the 4 subscales of the AIQ was significant, *F*(2,179) = 12.46, *p* < 0.001, *R^2^* = 0.18. Draft round explained 15.5% of the variance, but visual spatial processing explained an additional 2.5% above and beyond the effect of draft pick. Lower scores on visual spatial processing were associated with a greater number of TO. Table 3 shows the trimmed model for this HMR analysis.

### Free throw percentage and AIQ

The overall model for FT% as a function of draft pick round and the 4 subscales of the AIQ was significant, *F*(2,177) = 3.25, *p* = 0.023, *R^2^* = 0.05. Draft round was not related to FT%, but learning efficiency was a significant unique contributor, explaining 3% past the nonsignificant variance explained by draft round. It is worth noting that both decision-making and learning efficiency had significant positive zero-order correlations with FT%, but only learning efficiency had a unique contribution after adjusting for draft round. Higher scores on learning efficiency were associated with better FT%. Table 3 shows the trimmed model for this HMR analysis.

### Player efficiency rating and AIQ

The overall model for PER as a function of draft pick round and the 4 subscales of the AIQ was significant, *F*(3,175) = 6.95, *p* < 0.001, *R^2^* = 0.107. Draft round and decision-making were both significant unique contributors, explaining 8.6% and an additional 2.1% of the variance, respectively. Higher scores on decision-making were associated with higher PER values, both in the regression model and in the zero-order correlations. Table 4 shows the trimmed model for this HMR analysis.

**Table 4:** Hierarchical regression of performance metrics as a function of draft pick and AIQ factors.

Step and predictor variables	*R^2^*	∆*R^2^*	*sr^2^*	*β*	SEB
PER					
Step 1	0.09^***^	0.09^***^			
Draft pick round					
Step 2	0.11^***^	0.02^*^			
Decision making			0.15	0.14	0.029
eFG%					
Step 1	0.03^†^	0.03^†^			
Draft pick round					
Step 2	0.10^***^	0.07^***^			
Decision making			0.27	0.27	0.001
Pass efficiency%					
Step 1	0.04^*^	0.04^*^			
Draft pick round					
Step 2	0.07^***^	0.03^*^			
Decision making			0.18	0.18	007

^†^*p* < 0.10, ^*^p < 0.05, ^**^p < 0.01, ^***^p < 0.001.

### Effective field goal percentage and AIQ

The overall model for eFG% as a function of draft pick round and the 4 subscales of the AIQ was significant, *F*(3,176) = 6.71, *p* < 0.001, *R^2^* = 0.103. Draft round pick and decision-making were both significant unique contributors, explaining 3% and an additional 7% of the variance, respectively. While reaction time and decision-making both had significant positive zero-order correlations with eFG%, only decision-making had a unique contribution after adjusting for draft round. Higher scores on decision-making were associated with better Effective Field Goal percentages. Table 4 shows the trimmed model for this HMR analysis.

### Pass efficiency and AIQ

The overall model for Pass Efficiency, defined more commonly as assist to turnover ratio, as a function of draft round and the 4 subscales of the AIQ was significant, *F*(3,176) = 4.51, *p* = 0.004, *R^2^* = 0.07. Draft round and decision-making were both significant unique contributors, explaining 4% and an additional 3% of the variance, respectively. Higher scores on decision-making were associated with higher pass efficiency values. Table 4 shows the trimmed model for this HMR analysis.

## Discussion

Through the use of an empirically-validated and reliable measure of sport-specific intelligence, the role of cognitive abilities was assessed vis-à-vis the success of elite basketball players. Historically, NBA draft strategy has prioritized college basketball performance, athleticism, and anthropometry, in the absence of relevant data surrounding players’ cognitive functioning. However, when the evaluation of prospect potential is limited to these factors alone, there is room for improved prediction. The inclusion of AIQ data in the current study illustrates that the assessment of sport-specific intelligence contributes a modest but potentially important piece of the puzzle in the NBA draft process. Modest findings are often the norm in this area (Bergkamp et al., 2019), but offer the hope that there are opportunities to increase predictive power in the future, whether through better measurement, identifying other factors, or important interactions between factors.

Players drafted into the NBA were shown to possess higher scores across all AIQ factors when compared to their non-NBA counterparts (i.e., G League, International). Further, statistically significant differences were found between these two groups in 3 of the 4 broad categories that compose the AIQ (i.e., reaction time, decision-making, and learning efficiency) as well as the Full Scale AIQ Score. Additionally, drafted NBA players had significantly faster reaction times than either the non-NBA or undrafted NBA players. The effect sizes associated with these differences are generally small, but are consistent, both across the measures and with other studies that have attempted similar analyses for qualities such as anthropometry/physical skills and NBA performance (Cui et al., 2019), adolescent motor and anthropometric variables and subsequent soccer performance (Honer et al., 2017), as well as cognitive abilities and performance in professional baseball (Bowman et al., 2021). Thus, not only do drafted NBA players possess greater physical capabilities compared to non-NBA players (Cui et al., 2019), they also tend to have greater cognitive capabilities, connoting additional advantage.

Interestingly, differences were also found between undrafted players who ultimately made it to the NBA and those who did not. For each of the 4 broad factors, scores were higher for the undrafted NBA players; however, these differences only achieved statistical significance for the learning efficiency factor. Considerable NBA Combine data on physical tests and anthropometry indicate that undrafted players tend to have lower scores and smaller physical measurements in multiple areas (Cui et al., 2019). Thus, players who do not possess these physical traits and abilities necessary to be initially selected in the NBA draft, but ultimately make it to the NBA, must have some other features that help them make it to the league. Our data offers the possibility that cognitive abilities may play a role in that compensatory action. For instance, if a less physically gifted player possesses superior learning efficiency and can learn and recall game information, technique, and strategy at a higher level than others, this may contribute to his success. Further research is recommended to better identify the pattern of physical traits and abilities and cognitive capacities that help this group of players exceed expectations.

Looking specifically at players who have made it to the NBA, there was a significant correlation between PER and decision-making in particular, and the 4 factors of the AIQ accounted for a statistically significant amount of variance in this metric beyond draft round in a hierarchical multiple regression analysis. Similarly, both decision-making and visual spatial processing were significantly correlated with eFG%, and the 4 factors of the AIQ again explained a significant amount of variance in this statistic beyond draft round. In fact, the AIQ was more predictive of eFG% than draft placement. Significant relationships were also found between AIQ factors and the NBA statistics of FT%, Pass Efficiency, and TO, although not points per game.

Taken as a whole, the findings from this investigation suggest that cognitive ability may be a differentiator between elite basketball players who make it to the NBA and those who nearly make it to that level. Once players rise to that highest level of play, there may be greater parity, in terms of cognitive processing, just as there seems to be for physical ability. However, there appear to be certain cognitive factors that correlate with greater success on the court. Specifically, players’ decision-making may help them in multiple facets in the game, as reflected with the significant correlations with PER, eFG%, FT%, and marginal significance with Pass Efficiency. For instance, a strength in two-option decision-making may enable a player to make the right read in pick and roll or pick and pop plays. Similarly, the ability to quickly scan the floor for important information and details could help a player locate an open teammate when passing to an off-ball screening action or identify opposing players as he backpedals on defense.

There are also clear bases for the significant correlations found between visual spatial processing and reaction time and factors like eFG% and turnovers. For instance, strengths in visual spatial processing may impact a player’s ability to find efficient routes in transition with or without the ball. It could also help them maintain proper floor spacing. Additionally, a faster reaction time could help a player get a shot off faster under pressure. Each of these advantages could lead to better shot opportunities and a decreased likelihood of turning the ball over.

As with all research, there were limitations in the current study. Although there certainly appears to be evidence of predictive utility of the AIQ in professional basketball, without a direct comparison with this sample, we cannot know for sure whether one set of cognitive measures is significantly better than another. Additionally, the inclusion of other metrics, such as college performance statistics, anthropometric measurements, physical tests, and personality inventories may also explain some of the variance in the dependent variables considered.

Future researchers should seek to replicate and build on the findings herein. There is still much variance in performance left unexplained, allowing for models with even greater predictability to be determined. With a larger data set, this could potentially be done by comparing which of the 10 *subtests* of the AIQ play the largest roles in NBA performance (as opposed to only the 4 broad factors). This was not done in the current study in order to minimize the risk of Type I error. Closer analysis of the predictive validity of each subtest could help tailor cognitive assessments as well as interpretations of findings to specific positions, as certain intellectual abilities may be more or less impactful depending on the position.

To some degree, one limitation of this study was that it included a sample composed of only NBA basketball prospects/players. With comparisons being made only among such an elite group of basketball players, range restriction may be an issue, just as it is for physical skills and attributes (Bergkamp et al., 2019). Indeed, consistent with such other findings in evaluating predictors of metrics in professional sports where there is known to be a restricted range, the effect sizes of the differences and relationships in our study are generally small. Thus, future research should be undertaken to investigate the relationships between the cognitive abilities measured by the AIQ and performance in basketball for a more diverse group of players, such as those ranging from Division I to Division III in NCAA Basketball.

Finally, the dependent variables analyzed in the current study appear to adequately represent multiple aspects of NBA performance. However, future research may also benefit from the inclusion of other NBA metrics, such as Box Plus/Minus (BPM), Value over Replacement Player (VORP), and others. Our initial effort to explore these relationships focused on individual level measures, but it is worth considering including other common metrics to measure NBA performance more comprehensively. Although BPM and VORP are highly correlated with PER, their inclusion may lead to a more nuanced understanding of the impact of cognitive abilities in the NBA.

In the end, prior college basketball performance as well as measures of athleticism and anthropometry will undoubtedly continue to be weighed heavily in the talent identification process for the NBA – as they should be. However, the current findings suggest that, after likely controlling for the impact of college performance, athleticism and anthropometry captured by the draft process, differences in cognitive abilities contribute a unique, modest piece of the puzzle for NBA prospects as they reach the highest echelons in basketball.

## Data availability statement

The raw data supporting the conclusions of this article will be made available by the authors, without undue reservation.

## Ethics statement

Ethical review and approval was not required for the study on human participants in accordance with the local legislation and institutional requirements. The patients/participants provided their written informed consent to participate in this study.

## Author contributions

SH assisted with literature reviews, statistical analyses, manuscript writing, and editing. DT assisted with literature reviews, manuscript writing, and editing. RB and JB assisted with statistical analyses, manuscript writing, and editing. All authors contributed to the article and approved the submitted version.

## Conflict of interest

SH was employed by Strength Coach Scott, LLC, which is not affiliated with the AIQ. JB was employed by Athletic Intelligence Measures, LLC.

The remaining authors declare that the research was conducted in the absence of any commercial or financial relationships that could be construed as a potential conflict of interest.

## Publisher’s note

All claims expressed in this article are solely those of the authors and do not necessarily represent those of their affiliated organizations, or those of the publisher, the editors and the reviewers. Any product that may be evaluated in this article, or claim that may be made by its manufacturer, is not guaranteed or endorsed by the publisher.

## Footnotes

^1^Differences in df reflect the loss of AIQ data from a handful of players randomly distributed across the larger data set.
